# The digital transformation of healthcare - An interview with Werner Dorfmeister

**DOI:** 10.1007/s12525-021-00476-1

**Published:** 2021-06-15

**Authors:** Rainer Alt, Hans-Dieter Zimmermann

**Affiliations:** 1 Information Systems Institute, Leipzig University, Grimmaische Str. 12, 04109 Leipzig, Germany; 2 Institute for Information and Process Management IPM, School of Management, Eastern Switzerland University of Applied Sciences, Rosenbergstrasse 59, 9001 St. Gallen, Switzerland

**Keywords:** Digital transformation, Healthcare industry, Digital patient pathways

## Abstract

In this interview, Werner Dorfmeister, a Vice President at DasLab and a former Global Sales Director at Karl Storz, reports on the current developments of digital technologies in the field of medical instruments and services. It is a sector where digitalization affects all processes within hospitals as well as pre- and post-surgical activities. Due to the many actors involved, who use different information systems, and the strong regulation of the entire healthcare sector, crafting digital solutions is complex, but nevertheless promising and necessary. Among the key areas mentioned in the interview are telemedicine, hospital and patient management and a sector-wide digital infrastructure. Digital platforms are described as important enablers to address current inefficiencies and to leverage future potentials towards patient-orientation, such as digital patient pathways.

## Background information

Werner Dorfmeister was Global Sales Director at Karl Storz SE & Co. KG, a global producer of medical devices headquartered in Tuttlingen, Germany. The company was founded in 1945 and employs about 6′000 people today with annual revenues of approximately two billion Euros. Starting with the invention of the endoscope, Karl Storz has a long tradition in pioneering innovations in the fields of classical, heart and neuro surgery as well as gynecology and otolaryngology. The company is one of the largest producers of surgery instruments and has constantly enhanced its product portfolio with electronic devices and conceptualizing entirely integrated operating rooms. In March 2021, Werner joined DasLab GmbH, a German start-up company that focuses on the digitalization in the field of laboratory medicine. As Vice President Business and Partnerships, he is not only involved in the diagnostics of the Corona pandemic, but also investigates perspectives related to the “digital path” among laboratories, patients and healthcare organizations such as hospitals, physicians and insurance companies. Before joining Karl Storz, he was responsible for global solutions and commercial functions at the B2B services firm DXC.technology, which is a spin-off from Hewlett Packard Enterprises where Werner has worked in various positions since 2008. Prior to this position, he was sales manager at VMware and Microsoft. Werner has a degree in management with an emphasis in business informatics from the Donau University Krems and an MBA in media management from St. Pölten University of Applied Sciences in Austria. Since 1998, he is also a visiting professor at Donau University Krems and since 2014 he is a member of Electronic Markets’ advisory board. The pictures show him on the left followed by the interviewers Rainer Alt and Hans-Dieter Zimmermann.

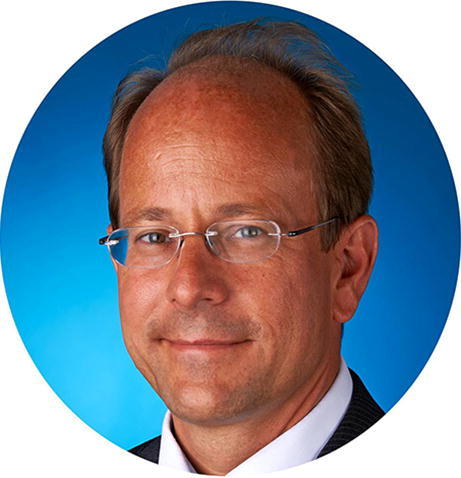

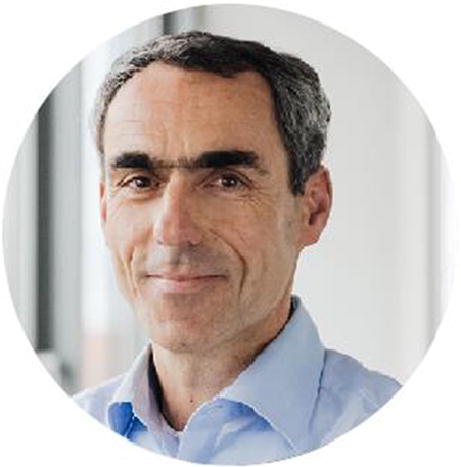

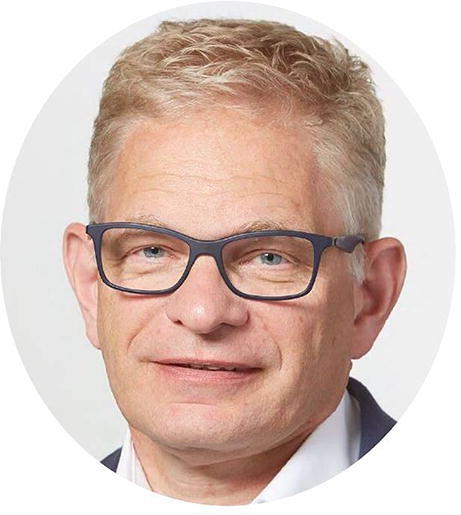







## How do you see information technology in medical and laboratory businesses?

Information technology has become an important element of medical products and a pillar in itself. For example, Karl Storz has seen a journey from a manufacturer of physical operation instruments to various information systems such as surgical information systems, telemedicine or systems for scheduling operation teams. This applies to surgical activities but also to the associated patient data-processing and accounting, which have been important issues for the last twenty years at minimum. This field applies to storage of patient data records and the billing of medical services. It is also visible in the organization of most hospitals, which are divided in a medical and an accounting department. Digital services are a broad and rather new segment, which reaches from aseptic displays to the illumination in operation rooms. For example, studies have shown that in pediatric surgery the outcome is positively influenced if the child is able to watch video clips during surgery or if the room is illuminated in attractive colors. Increasingly we also see initiatives regarding pre- and post-surgical activities, for instance, when surgery teams in hospitals are allocated to post-surgical treatments, such as physical rehab and kinesiatrics. 

## What is the role of digitalization for hospitals?

Adopting information technology and leveraging the potentials of this digital revolution has become a necessity for every hospital. Recall that large volumes of data accrue in these organizations and that the entire system is still rather paper-based. At the same time, regulatory and market pressures have increased, which forces numerous hospitals to fight for survival. Tapping new sources of revenue has also proved to be difficult since many potentials are simply not feasible due to legal restrictions. If hospitals aim to monetize their wealth of data, they have difficulty in doing this. I could imagine many situations where providing medical data for research purposes would be helpful. Let me give you an example: endoscopes always produce video data, but these are not recorded beyond some still images which get extracted. It would be beneficial using the entire video for evaluation and other purposes. However, due to the existing remuneration mechanisms as well as to the complexity of the processes, hospitals today actually earn money primarily on surgical operations and are little motivated to offer such services.

## Is the problem mainly a regulatory issue?

This is one aspect amongst others. Hospitals are still dominated by the mechanical tasks that are associated with surgery. While this is undoubtedly important, there are complementary and even critical issues that need to be considered. Let me distinguish three areas: first, there are activities around surgery, such as supporting the surgeon in the room. This could be done not only with intelligent equipment in the room, but also with telemedicine. Telemedicine for itself has two facets: on the one hand, you have the virtual relationship between the doctor and the patient. On the other hand, you might also have the relationship between doctors. How will a physician learn to perform surgery? Since surgery is a mechanical task, an experienced physician will typically assist the beginner and this could be “virtualized” to a certain extent. Second, there is the entire field of hospital management, which includes the assignment of surgeon teams and their equipment as well as the field of patient management. We are expecting substantial benefits from digitalization here. Finally, there is the infrastructure, where we are facing new challenges with cybersecurity. As you might have heard from various attacks on hospitals, the situation is critical and in comparison, other industries are clearly advanced. This is due to structural problems, since covering the costs for this infrastructure is difficult and not compensated by health insurance companies.

## So what could hospitals learn from other industries?

Although information technologies made early inroads in the healthcare sector, the digital transformation has merely started and the sector lags when compared with other industries. Much progress would be feasible by adopting existing principles that are known as best practices in supply chain management, marketing or customer relationship management. In fact, most activities are still limited to the individual organization and much more networking would be conceivable in the future. I see digitalization as an important enabler in this respect.

## Does this also apply to doctor’s offices?

Absolutely - over the last two decades, many doctor’s offices have introduced information systems. This means that digital know-how is at least partly available, but the integration of doctor’s information systems with other institutions and the standardization of processes as well as data is still in its infancy. In this respect, the pandemic we experienced in 2020 has given some impetus to the entire topic of digitalization and we saw a stronger use of telemedicine. However, the regulatory problem surfaced again since these virtual services could not be charged adequately, which stifled the motivation of physicians and hospitals to make more use of these potentials.

## What is the status quo regarding digital standards?

The time I started my professional career dates back to the emergence of electronic data interchange (EDI). Standards from the EDI world in the 1980s and 1990s are HL7 (Health level seven) for the exchange of patient-related data, so called electronic health records (EHR), which are present and were updated to adopt XML with FHIR (Fast healthcare interoperability resources). Another standard is DICOM, which stands for digital imaging and communications in medicine and allows the exchange of radiologic and endoscopic pictures. In addition, organizations that operate exchange infrastructures, such as Gematik in Germany, evolved. Although these solutions are valuable, they are message-oriented and much more is feasible with digital platforms, assistants and services today.

## Is this a call for digital platforms and electronic markets?

Digital platforms are vital for many coordination processes. For example, think about how recommender systems support the search for doctors and clinics. This area is still at the beginning with plastic surgery being the pioneer. Regarding the emergence of such platforms, we need to differentiate between national and global ones. There are national initiatives, such as the telematics infrastructure and the electronic health record driven by Gematik in Germany, which almost inherently face the problem of being incompatible with other national solutions. This is different with cross-national initiatives like the European Commission’s eHealth network and in particular with global initiatives. At present, most big tech or GAFAM businesses like Google, Amazon, Facebook, Apple or Microsoft are present globally. They run large programs in the field of digital healthcare and also include international standards such as FHIR. This allows much more efficiency in exchanging data between many organizations. For example, companies such as Johnson&Johnson are among the largest providers of medical care data.

## How do you consider the impact of such platforms?

These platforms are enablers in offsetting the potentials in supply chain and customer – or as some say, patient – relationship management. Let me give you three examples. First, it’s about optimization of hospital operations, such as the management of devices in surgery and intensive care as well as the maintenance and localization of these devices or instruments. For example, potentials from electronic procurement could be leveraged, if surgeons would not purchase different instruments by themselves, but rather if the entire procurement process would be standardized. Second, we could coordinate all activities around the patient by collecting data from patient-related activities. Instead of documenting all activities on paper, for example if an endoscope fails during a surgery, sharing the data with manufacturers would enable them to get involved and to learn from the adverse event. It would also help to assess legal charges and reimbursements. There are also developments to assess surgery results in a longer time frame. In the US, improvements after the treatment are monitored by continuously asking patients about their condition for six months after surgery. Finally, there is the area of digital patient pathways: this is a radical new concept and features important changes, compared to how data is exchanged today. At present, patients see many institutions that all have their proprietary and cumbersome integrated systems. By using modern technology, such as platforms and apps, I see much more progress in the patient’s interest.

## Could you elaborate on this concept of digital patient pathways?

I give you an example: when seeing the physician, you typically receive a doctor’s letter that has been printed from a computer system and that you take to the next “health worker”. This paper-based scenario is not only inefficient, but also fails to include much data which is available today. Data could be shared using established standards between the involved organizations and more data could be provided in this process. One example is data from your fitness tracker or your smartwatch, which collects valuable data that is currently not shared with your physician. The digital patient pathway covers all steps from diagnosing the disease until post-surgical activities and treatments, including supporting doctors and surgeons during the surgery itself. Based on data created throughout surgery and treatments, these activities could be planned, communicated, and coordinated, including the necessary facilities, rooms and people. At present, several processes are not integrated at all although some systems and respective services are already available. For example, apps for guiding patients during aftercare are not widespread so far as, and I mentioned it before, hospitals do not receive any remuneration for using them.

## Do you expect a major transformation of how healthcare is done and organized?

Yes, healthcare in the future will be more patient-oriented and organized in a more structured and transparent way among many participants. My hope would be that hospitals are in a better position in the competition and that they are able to learn from one another. Comparing and benchmarking should not be confused with enforcing cost constraints, but should be a source for improvement. In much the same way, the existing resources in hospitals could be used more efficiently. Recall that many operating rooms and many medical devices are only partly used, some of them with only 20 to 30% of their available capacity. Digital platforms could improve this allocation task and help to induce more flexibility in the system. Instead of employing all doctors internally, hospitals in the US are using the model of attending doctors much stronger than hospitals in Europe.

## What about the treatments themselves?

Of course, digitalization needs to have a positive impact on the precision and quality of treatments. This ranges from using artificial intelligence in the diagnosis stage to the surgery itself. We see interesting applications of artificial intelligence technologies, which mainly support the physician when diagnosing a disease or when screening documents. Robotics and three-dimensional modeling are making inroads in the surgery today in some bigger hospitals. This could allow surgeons to train the intervention before executing it on the patient and to use augmented reality technologies during surgery. This development could be linked with telemedicine, which would make the knowledge of specialists worldwide available in operating rooms. Finally, I hope that digitalization will also include the pharmaceutical sector, which is often separated from healthcare. Many of the potentials for digitalization in this sector have a strong impact on healthcare, for example, addressing product safety, approval, or optimization of test procedures for drugs.

## How is the sector prepared to handle personal data?

This is a critical and sensible issue. On the one hand, we see the need to exchange data among many institutions, on the other hand, we know that it inherently bears many risks for data privacy. My hope is that the sector makes progress in increasing cybersecurity measures and that data may not only be exchanged, but also collected and analyzed for research purposes. As already mentioned, the medical sector is one of the largest providers of data and we should be able to exploit the vast amounts of data from surgery and treatments. Scandinavian countries are leading in Europe regarding the analysis of clinical disease data to combat various diseases. This allows large pools with health data to be used to follow the development of certain diseases and other purposes. For example, Denmark has established a digital data pool which contains medical and life data of the entire population. It has already started in the 1990s to collect medical records since 1945 and constantly pushed for standardizing digital health records. Researchers worldwide now have the opportunity to analyze this wealth of historical data, which includes the types and courses of diseases. This could yield new insights in many critical diseases such as cancer and reconciling the data with other data sources, such as socioeconomic data like place of residence or profession, could reveal further findings. Finland is also working on such a database, but, unfortunately, the remaining European countries are not very active in this direction.

## How are big tech companies participating in this endeavor?

Indeed, the GAFAM companies have recognized the opportunities and are very active in this domain. While they all support FHIR, they pursue different strategies: Microsoft focuses on platform access for doctors as well as hospitals, and Google and Apple on various applications for healthcare and wellness. The question arises: which platform has the potential to act as the gatekeeper for health-related issues? Is data with the big tech companies, is it with the customers (i.e. the patients) or is it with other actors in the healthcare market? As mentioned, Johnson&Johnson has already established one of the largest clouds for medical data. Almost automatically, the tech companies have an advantage, since the use of their devices (e.g. a smartwatch) will imply that our data is stored in the provider’s cloud. Users have only little possibilities to store data in a place of their own choice. Since the big tech companies are non-European companies, a European cloud initiative would be a valuable approach. However, we are only at the beginning and it will be difficult to “lure” patients away from the big tech platforms. In certain situations, patients simply will not care about where their data is stored. Imagine having cancer, you will not really care where you put the check mark. So far, there is only little ethical discussion on this issue at the research level, and almost none at all at the commercial level.

## Do you see implications of the Corona pandemic?

In fact, the pandemic has brought many aspects of digitalization to the table. Patients, healthcare workers and public authorities have recognized that structural changes are necessary in the healthcare sector. In particular, this refers to the regulatory issues mentioned earlier, which impede many innovations, as well as to how staff and activities in the medical sectors are remunerated. In particular, digitalization requires infrastructural and standardization activities, which not only need public attention, but also a joint effort of the many actors in the pharmaceutical, medical and wellness sectors. From my present role, I see the situation in the laboratory industry similar to the situation in the hospital sector. In fact, I feel that digital platforms are even more relevant here due to inefficiencies in the laboratory market. Finally, we have to support doctors in dealing with data security issues, as they are often with one foot in jail when exchanging personal health data with colleagues.

## What role do you see for academic research?

Medical businesses have a strong tradition in conducting research activities in cooperation with universities. However, this often refers to the medical as well as the engineering disciplines. For sure, we also have to strengthen the collaboration for the different aspects of digitalization I mentioned above. These include computer science with software engineering in general, and imaging methods in particular, as well as the areas of telemedicine and the field of digital patient pathways. I am convinced that the topics of the Electronic Markets journal, meaning digital platforms and networked business, are vital for our future strategies in healthcare.

Dear Werner, thank you for this interview.

